# Effectiveness of peer support interventions to improve mental health outcomes after miscarriage: a systematic review and call for high-quality evidence

**DOI:** 10.1136/bmjopen-2025-109556

**Published:** 2026-06-24

**Authors:** Leanne Burton, Juanita Charles, Mary Gemma Cherry, Rhiannon Corcoran, Shaima Hassan, Ruaraidh Hill, Sarah Little, Michelle Maden, Helen Mulholland, Elizabeth Perkins, Pauline Slade, Selina Wallis, Paul Marshall

**Affiliations:** 1 Department of Primary Care and Mental Health, Institute of Population Health Sciences, University of Liverpool, Liverpool, UK; 2 The Miscarriage Association, Wakefield, UK; 3 Liverpool Reviews and Implementation Group (LRiG), Department of Health Data Science, Institute of Population Health Sciences, University of Liverpool, Liverpool, UK; 4 Independent Researcher (Public Advisor), Merseyside, UK

**Keywords:** MENTAL HEALTH, Pregnancy, Health Services, Psychosocial Intervention, Postpartum Women

## Abstract

**Abstract:**

**Objectives:**

Peer support is being integrated into the new maternal mental health services in England to further the development of the recovery approach in relation to loss. Psychological support after miscarriage (pregnancy loss prior to viability) is often overlooked, despite significant psychological morbidity. This systematic review explored the effectiveness of peer support interventions to improve mental health outcomes after miscarriage.

**Design:**

Systematic review

**Data sources:**

A comprehensive systematic search across nine databases (MEDLINE, CINAHL, APA PsycINFO, Web of Science (all databases), EMBASE, CENTRAL, LENS.org, British Nursing Index and Health Management Information Consortium) was conducted in June 2025. Grey literature was identified through website searching, contact with topic experts and a national call for evidence.

**Eligibility criteria:**

Study designs with a quantitative evaluative component or mixed-methods studies reporting effectiveness were eligible if they involved women and/or partners who had experienced miscarriage and been offered a peer support intervention. Any peer support versus control (no treatment, wait list and usual care) or peer support versus another psychosocial intervention was eligible for inclusion. Studies that report any of the following broad groups of outcomes (whether validated measures or by self-report) were eligible for inclusion: (a) personal recovery, (b) mental health recovery, (c) health service use and (d) social outcomes.

**Data extraction and synthesis:**

Two independent reviewers used standardised methods to search and screen for eligible studies.

**Results:**

Of the 4342 titles screened, 100 potentially relevant full-text papers were retrieved and screened resulting in seven randomised controlled trials and two controlled trials identified across 10 papers. Of these, seven did not evaluate a peer-led intervention, one reported only on women who had experienced pregnancy loss later than 24 weeks gestation, and one reported a peer support intervention for those who experienced pregnancy loss at any age of gestation but did not disaggregate data for those who experienced miscarriage. Thus, no studies were eligible for inclusion. This indicates a significant gap in the current literature. The inconsistencies and limitations in existing research approaches are explored in detail.

**Conclusions:**

This systematic review has identified an evidence gap as there is currently no robust evidence for the effectiveness of peer support interventions after miscarriage. Given the drive for the inclusion of peer support in new maternal mental health services, there is therefore a need for targeted intervention research to provide reliable evidence to determine effective peer support interventions for this population.

**PROSPERO registration number:**

CRD42024518248

**STRENGTHS AND LIMITATIONS OF THIS STUDY**

- Study design that incorporated a thorough literature search, including grey literature searching and a call for evidence, to allow for a detailed investigation of current evidence.
- The use of a comprehensive systematic review methodology provides a credible case for the ‘evidence gaps’ in peer support after miscarriage, increasing awareness as well as serving as a call to action for the development and evaluation of such interventions.
- Using the review methods, studies in the topic area can be categorised, informing important questions and discussions about how ‘effectiveness’ of peer support can be measured.
- The lack of studies reporting suitable data meant that no further analysis or conclusions could be drawn about the effectiveness of peer support for those who have experienced miscarriage.

## Introduction

 Miscarriage, or early pregnancy loss, is estimated to occur 23 million times worldwide yearly, affecting 1 in 10 women in their lifetime.^1^ To date, there is no standardised consensus definition of miscarriage, with definitions differing from country to country and varying by upper gestational age and pregnancy viability. Miscarriage is most often defined as pregnancy loss prior to viability. However, viability also varies and differs between a clinical and legal perspective. The WHO defines miscarriage as pregnancy loss prior to 28 weeks gestation,^2^ whereas the Royal College of Obstetrics and Gynaecologists in the UK defines miscarriage as pregnancy loss <24 completed weeks gestation.^3^ In contrast, the American Society of Reproductive Medicine uses a lower viability threshold of 20 weeks.^4 5^

International literature suggests the presence of a significant psychological morbidity associated with miscarriage, for both women and their partners.^6 7^ In a study investigating the type and severity of emotional distress in women after miscarriage, 28% met the criteria for probable post-traumatic stress disorder (PTSD), 32% for anxiety and 16% for depression at 1 month, and 38%, 20% and 5%, respectively, at 3 months.^8^ According to a review by Farren *et al*, anxiety is the most common and persistent psychological disorder after a miscarriage.^9^ Similarly, in women who have experienced miscarriage, the relative risk of having a diagnosis of depression is reported to be 3–4 times higher than in a control group of non-pregnant women.^9^ Additionally, 1 in 12 partners was found to have long-term symptoms of PTSD after miscarriage, demonstrating the impact on both parents.^10^

While healthcare routinely focuses on the physical effects of early pregnancy loss, psychological support appears to be significantly less well developed and less considered.^11^ The UK’s 2023 Pregnancy Loss Review highlights current insufficiencies in mental health support after miscarriage and makes recommendations for offering psychological support, where necessary, to both parents.^12^

The National Institute for Health and Care Excellence recognises that grief following miscarriage is ‘comparable in nature, intensity and duration’ to grief reactions in people suffering other types of major loss.^13^ Private grief and misconceptions surrounding grief after miscarriage can lead to women and their partners feeling at fault or managing alone.^14^ Psychological distress can be exacerbated by feelings of shame, blame and stigma that often accompany early pregnancy loss.^15 16^ Marginalisation and the use of negative language around miscarriage by both healthcare professionals and within wider society can have long-lasting psychological impacts and result in increased intensity and duration of grief.^17^ A recent systematic review exploring how people ‘story’ their miscarriage experience highlighted a lack of social recognition, which disenfranchises a parents’ grief; people around the bereaved parents may lack an understanding of the significance of the loss, which may increase feelings of isolation, with parents not receiving the social support that they need.^18 19^

Social support, a multidimensional concept, is typically measured in terms of either the structure (number of relationships) or the functions (eg, informational, instrumental and emotional) of social networks.^20^ Social support plays a protective role in mental health^21^; inadequate social support is a significant predictor of postpartum depression.^22^ Research suggests that social support in the bereaved population can enhance emotional well-being and reduce psychological distress (eg, diminished anxiety and depressive symptoms).^23^ In other populations, such as those managing symptoms of diabetes,^24^ in patients with heart failure^25^ and for those undergoing cancer treatment,^26^ evidence suggests that support received from those who have had similar experiences, known as peer support, can enhance mental health and well-being.

Peer support is defined as *“the provision of emotional, appraisal and informational assistance by a created social network member who possesses experiential knowledge of a specific behaviour or stressor and similar characteristics as the target population”* ^27^. Peer support relates to support, hope and encouragement provided to those who share similar experiences.^28^ Several conceptual models explain how peer support might benefit those with mental ill-health.^27^ Peer support is hypothesised to involve building social relationships that have reciprocal influence on health and well-being, as shown in patients with various mental illnesses.^29^ Peer support strategies may also offer emotional and social benefits for both service users and caregivers.^30^ Dennis’ conceptual framework described four overlapping mechanisms by which peer support may offer benefit to service users: (i) reducing feelings of isolation and inferiority and a positive impact on ‘bad’ habits (direct effect), (ii) buffering the harmful effects caused by stressors (buffering effect), (iii) improving self-efficacy and (iv) guiding and encouraging cognitive restructuring (mediation effect).^27^

The evidence for psychological support services, particularly peer support interventions, to improve mental well-being after miscarriage is scarce.^31 32^ A Cochrane systematic review published by Murphy et al. in 2012^31^ identified only six randomised controlled trials (RCTs) that examined non-pharmacological interventions to improve the mental well-being of women who had experienced miscarriage, none of which were peer support interventions nor incorporated any elements of peer support. This lack of evidence is notable given the potential relevance of peer support for individuals experiencing grief following miscarriage, where shared lived experience may provide emotional validation, reduced social isolation and address gaps in formal mental and physical healthcare provision.^30^

Owing to the methodological limitations of the included studies, the authors concluded that the available evidence was insufficient to demonstrate the effectiveness of the offered psychological support. While Murphy’s review focused on RCTs, cohort studies and clinical trials suggest that psychological interventions and specific support interventions could improve mental well-being in women who experience pregnancy loss. Despite available data from other types of pregnancy loss experiences, which describe the impact of psychological support,^33^ information on the influence of peer support specifically for those who have experienced miscarriage is limited.^34 35^ The lack of evidence is particularly significant given previous studies suggesting that women who experience miscarriage value support from those who have had similar experiences.^36 37^ This review therefore focuses specifically on early pregnancy loss because the care and support landscape differs substantially by gestational stage. Individuals experiencing early loss are less likely to access specialist maternity or bereavement services and often report limited social recognition and formal follow-up.^18 19^ In this context, peer support may play a distinct role that is not directly comparable to support needs following later-stage pregnancy loss.

Despite the recognition of the psychosocial impacts of miscarriage, the nature, availability and effectiveness of peer support interventions in this population remain poorly characterised. This review therefore seeks to address this gap by systematically examining the available evidence on peer support in the context of early pregnancy loss.

The objectives were (a) to identify, summarise and assess the quality of the available evidence base for peer support interventions for women and their partners after miscarriage, (b) provide an overview of the characteristics, nature and diversity of interventions currently available and (c) explore the evidence in terms of outcomes of interest including (a) personal recovery, (b) mental health recovery, (c) health service use and (d) social outcomes. The review aimed to focus on interventions that had been subjected to evaluation in relation to their clinical and/or cost effectiveness.

## Methods

The systematic review was conducted in accordance with Joanna Briggs Institute (JBI) standards^38^ and reporting follows the Preferred Reporting Items for Systematic Reviews and Meta-Analyses (PRISMA) guidance for systematic reviews.^39^ This systematic review has been registered in the international prospective register of systematic reviews (PROSPERO) database (CRD42024518248).

### Search strategy

A comprehensive search strategy was undertaken in the following databases: MEDLINE, CINAHL, APA PsycINFO, Web of Science (all databases), EMBASE, CENTRAL, LENS.org, British Nursing Index and Health Management Information Consortium. All database searches were undertaken in June–July 2024, with an updated search conducted in June 2025. Searches were limited by date and included only evidence obtained since 1990 when peer support was formally introduced as a service in mental healthcare. Both UK and international studies, written in English, were included, as elements of peer support provision in other healthcare/non-clinical settings may be transferable to the UK. Keywords and Medical Subject Headings were used to identify relevant studies. Key search terms included population terms (eg, miscarriage, pregnancy loss and spontaneous abortion), peer terms (eg, layperson, friend and mutual) and intervention terms (eg, networking, social support and peer group). Truncation and proximity operators were employed to increase the sensitivity of the search. References of all relevant publications were reviewed. Reference lists of relevant systematic reviews were searched (see online supplementary file 1 for full search strategies).

In identifying innovative and emerging practices, both in the NHS and third sector organisations, grey literature was searched, as it was likely that relevant literature would not be available in the peer-reviewed published domain. A broad approach was adopted to search for grey literature combining (a) Google Scholar search, (b) searching unpublished postgraduate theses via ProQuest Dissertation and Theses, (c) scanning relevant websites for relevant literature and (d) asking steering group members to identify topic experts, useful websites and organisations to contact that may have additional evidence. Searches for grey literature were undertaken in August 2024 and updated in June 2025.

Additionally, a call for evidence was promoted, targeting topic experts, stakeholders and peer support providers to identify any unpublished data/research in progress.^40^ The call for evidence was shared through expert networks, direct emails and targeted social media. Community groups and charities were contacted to identify materials in community-based collections. The call for evidence was approved by University of Liverpool Research Ethics Committee (ref: 14581) and was live from 1 October 2024 for 6 weeks.

### Patient and public involvement

Our study Public Advisory Group were involved in the design of the search strategy for this review, as well as in dissemination plans of this research.

### Study selection

Titles and abstracts were imported into Rayyan.ai (web-based platform designed for managing systematic reviews) for screening.^41^ A two-stage approach for screening was adopted. Stage 1 involved screening of titles and abstracts. Two reviewers (LB and EW) independently screened titles and abstracts against the inclusion criteria to identify relevant papers for full-text review. Disagreements were resolved through discussion and consensus, with a third reviewer (PM) consulted when necessary.

### Eligibility criteria

Eligibility criteria are outlined below (table 1):

**Table 1:** Eligibility criteria for studies considered for inclusion in the review

	Inclusion	Exclusion
Participants	Parent(s) (ie, women and birthing people or their partners) with experience of miscarriage (loss of pregnancy up to 24 weeks gestation) who accessed peer support intervention(s) after miscarriage*	Studies conducted with a population who had experienced later pregnancy loss, stillbirth or infant loss, where it was not possible to separate miscarriage-related data (<24 weeks gestation) Peer support in other population groups (eg, grandparents)
Interventions	Peer support, defined as the existence of a community of common interest where people gather (in person or virtually by telephone or computer) to share experiences, ask questions, provide emotional support and gain self-help Interventions utilising a formal or professional facilitator, provided the role of the facilitator is to manage group interpersonal processes rather than provide counselling or any other psychoeducation	Interventions which are not peer support (i.e. any other form of psychosocial or psychoeducational support), including where offered as an adjunct to peer support. Peer support offered directly by family members (ie. partners and parents)
Comparator	Any peer support versus control (no treatment, wait list and usual care) or peer support versus another psychosocial intervention	
Outcomes	Studies that report any of the following broad groups of outcomes (whether validated or by self-report): (a) personal recovery, (b) clinical recovery, (c) health service utilisation and (d) social outcomes	Studies describing the use of an intervention in practice with no evidence of outcome evaluation Qualitative studies
Design	Study designs with an evaluative component (eg, any study design that reported within- or between-group comparative data, including studies with an independent control group, randomised controlled trials or single group pre-intervention–post-intervention studies) and mixed-methods studies reporting effectiveness	Conference abstracts, protocols and reviews

* We use terms such as ‘women’, ‘parents’ and ‘birthing people’ throughout this review. We recognise that not all individuals who experience pregnancy or miscarriage identify as women. Our goal is to be inclusive while reflecting the language used in relevant literature and by participants in peer support programmes.

Studies that clearly met the inclusion criteria, or in instances in which the relevance of the study was unclear, were taken forward to the next stage. Stage 2 involved screening of full texts against inclusion criteria for final inclusion in the review. Any discrepancies in the inclusion of abstracts or full-text articles were resolved by referral to a third reviewer (PM) and discussion with the project team.

## Results

**Figure 1:**
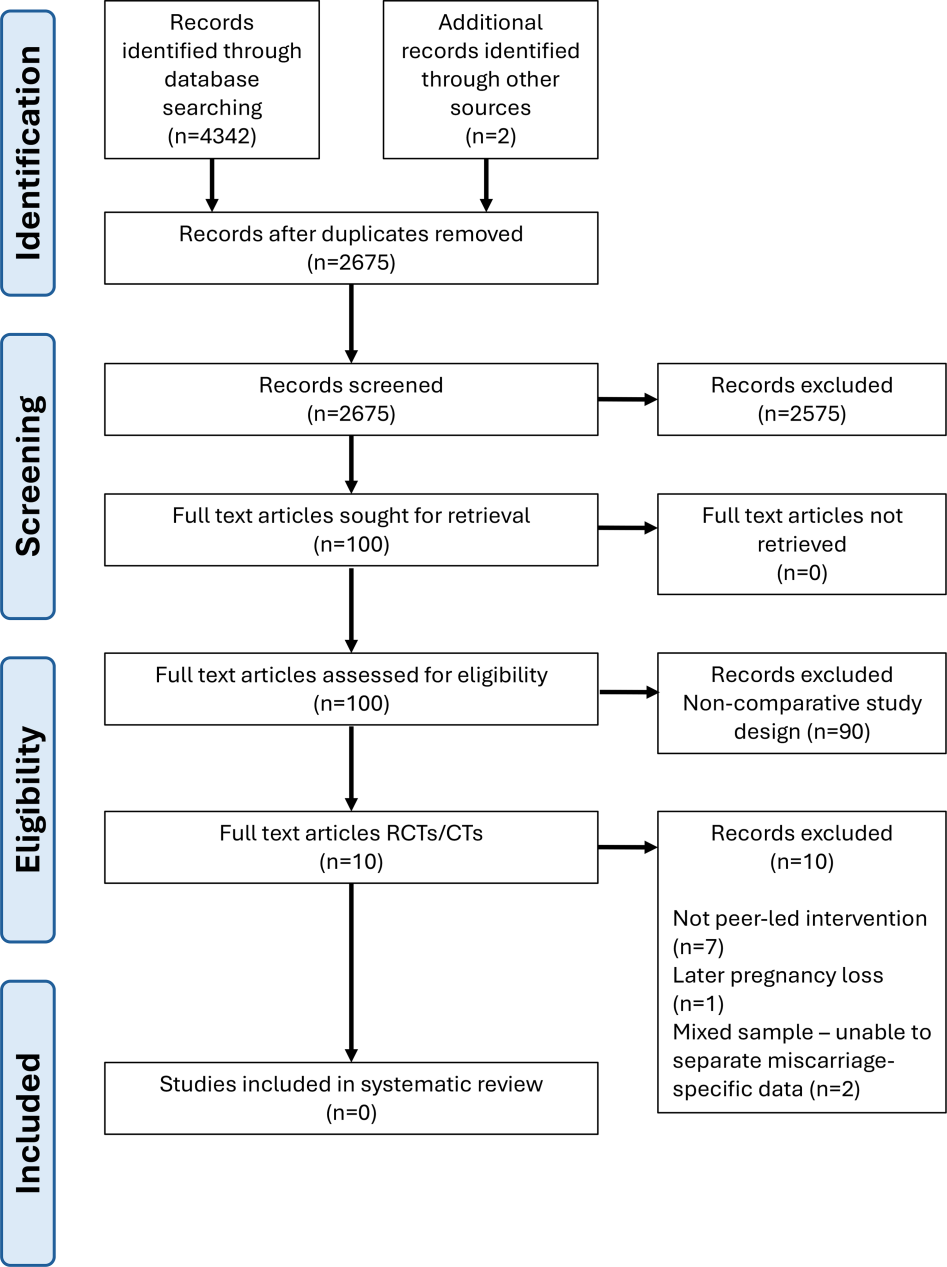
PRISMA flow diagram of study selection. Flow diagram illustrating the process of study identification, screening, eligibility assessment and inclusion in the systematic review, conducted in line with PRISMA guidelines. RCT, randomised controlled trial.

The combined database search retrieved 4342 records, leaving 2673 after duplicates were removed. Grey literature searches identified two other sources; therefore, 2675 records were screened by title and abstract. No RCT or other effectiveness trials identified met the inclusion criteria for this review (see figure 1 for the PRISMA flow diagram).

However, the review found seven RCTs^28 42^ and two controlled trials.^43 44^ Although they did not meet the inclusion criteria, they partially aligned with the scope of this review (online supplemental appendix 2). Of the effectiveness trials identified, seven were excluded because they did not evaluate a peer-led intervention.^4243 45^,^49^ Four of these reported interventions which were led by healthcare professionals,^43 45 47 48^ one was a self-management intervention,^44^ and two reported website interventions with lived experience elements.^42 49^ Both interventions had several components, including online forums^42^ and modules with reference to lived experience.^48^ As the lived experience elements were an adjunct to a multicomponent, complex intervention, these studies were also excluded. One intervention was excluded, as it was designed for women who had experienced pregnancy loss later than 24 weeks gestation.^45^ A final study did report on a peer support group intervention for those who had experienced pregnancy loss at any gestation; however, because the results from those who had experienced miscarriage were not disaggregated from others who had experienced later pregnancy loss and were not reported separately, this study was also ineligible for inclusion.^50^

Collectively, these studies highlight that although post-miscarriage support interventions were evaluated to some extent, peer support remains under-represented and insufficiently examined as a distinct intervention. This reinforces the gap identified in this review and underscores the need for rigorously designed studies focusing specifically on peer support after miscarriage, addressing the needs of both women/birthing people and their partners.

## Discussion

This systematic review was conducted to explore the effectiveness of peer support interventions in regard to personal and mental health recovery, health service use and social outcomes for parents who have experienced miscarriage. This review found no studies eligible for inclusion. The lack of research evaluating peer support interventions in this population is unfortunate considering the increased negative mental health implications that occur immediately following and years after a miscarriage.^8^,^10^

Maternal Mental Health Services were commissioned in 2021 to combine maternity, reproductive health and psychological therapy supporting women who have moderate to severe mental health conditions directly related to their pregnancy experiences.^51^ Peer support is a key component of this service offer.^52 53^ It is concerning that within a new service model, in which at least 50% of programmes employ paid peer support workers, there remains little evidence of their effectiveness.^51^ Despite the interest in health services for the introduction of peer support, it appears that, at present, practice precedes evidence. The failure to evaluate the effectiveness of peer support interventions for parents who experience miscarriage is therefore a significant omission for evidence-based practice. We hope that this review, in identifying this gap in the evidence base, will serve as a call to action for the development and evaluation of peer support interventions in this population. Going forward, it is important to address this evidence gap to ensure that effective peer support services are being designed and implemented to support parents who have experienced miscarriage.

### Mental health needs after miscarriage

Prior non-evaluative and qualitative studies have examined how people cope with grief following miscarriage and the benefits that may be derived from social support, support groups and other forms of aftercare.^37 54 55^ Such studies have shown that the support needs of those who experienced miscarriage may differ from other types of pregnancy loss (eg, later pregnancy loss and stillbirth).^36 37 54 55^ Later pregnancy losses tend to be more publicly acknowledged, whereas stigma surrounding miscarriage means that people often suffer in silence. Early pregnancy loss is both physical and symbolic; the loss of a child, reframing bodily autonomy and often identity.^56^ The ambiguity of early pregnancy loss can be difficult for non-peers to understand and validate.^55^ As such, miscarriage-specific support groups often centre around validating grief that society often does not recognise.

As services are increasingly intended to reflect the interests and priorities of service users, the extent to which the outcomes used to evaluate services within healthcare systems align with the outcomes valued by service users is questioned.^57^ The COMET Initiative database highlights ongoing work to develop a Core Outcome Set for mental health following early pregnancy loss, which may highlight key outcomes that are important to women and their partners and allow research to focus on outcomes deemed most important by key stakeholders.^58^ Consideration of such findings, in the absence of effectiveness research, could support the development of recommendations for future peer support interventions, with potential to incorporate the needs of service users in the intervention design stage utilising co-design approaches.

### Implications for future research

Whether peer support is effective in improving mental health outcomes for those who experience miscarriage currently remains unknown. The null results of this review, despite the increasing popularity of peer support in the perinatal mental health landscape, underpins our call to action for future research to build an evidence base to understand the effectiveness of peer support interventions in the miscarriage population including measures that are meaningful to both service users and healthcare providers.

### Measuring ‘effectiveness’ in peer support after miscarriage

In exploring the effectiveness of mental health services, including peer support, there remains a focus on clinical outcomes, such as symptomatology and social disability and service use (eg, readmission rates).^59 60^ This is despite an international shift towards recovery-oriented models of care in mental health.^61 62^ Repper and Perkins summarise the challenge: *“Traditional yardsticks of success – the alleviation of symptoms and discharge from services – are replaced by questions about whether people are able to do the things that give their lives meaning and purpose, irrespective of whether their problems continue and whether or not they continue to need help and support”* .^63^ This is particularly relevant in the miscarriage population, given reflections on the perpetual grief which follows early pregnancy loss.^64^

Research suggests that in the peer support landscape, effectiveness is often challenging to demonstrate using clinically based outcomes.^65^ A recent umbrella review exploring the effectiveness of peer support delivered by paid peer support workers with lived experience of mental health conditions found that many systematic reviews reporting effectiveness data reported no effect of peer support on a range of clinical outcomes.^66^ Most of the evidence regarding mental health symptom severity among those with mental health diagnoses or who were using mental health services suggested no effect, with the exception of perinatal depression and suicidal ideation.

Peer support was never envisioned as a substitute for clinical services and thus has different goals and outcomes than those of clinical services. As such, it is not methodologically sound to compare outcomes of peer support with those of clinical services or to measure such outcomes in the same way.^67^ Recent research has begun to demonstrate that peer support interventions may be less effective in changing clinical outcomes, at least in the short term, but more effective in employing recovery-oriented outcomes such as empowerment, self-efficacy and hopefulness.^68^,^70^ Cooper’s umbrella systematic review found seven reviews with meta-analysis reporting data on overall self-reported recovery, with five reporting significant improvements in recovery outcomes (eg, hope, empowerment, goal attainment and quality of life) in adults with mental health diagnoses including severe mental illness.^66^ Narrative reviews also indicated that peer support effectiveness can be demonstrated through improvements in recovery-oriented outcomes more so than clinical outcomes. While the outcome focus of our review of peer support interventions after miscarriage was broadened to encompass such outcomes, it appears that research in miscarriage peer support has not yet developed to embrace this way of thinking.

Given the recovery-oriented focus of peer support is to support individuals to live a satisfying, meaningful life, it may be that clinical outcomes are not the most appropriate indices by which to identify change. Recovery-oriented outcome measures, such as self-efficacy, empowerment and hopefulness, may better reflect the recovery values of peer support.^71 72^ Additionally, an alternative and potentially important measure in peer support may be that of ‘goal-based’ outcomes. Goal setting is often used in mental health recovery to support individuals to identify personally meaningful outcomes that become the target of treatment and support.^73^ Despite their individualistic nature, in that goals will differ from person to person, personal goals can and should contribute to outcome evaluation. Brief outcome measures such as the Measure Yourself Medical Outcome Profile (MYMOP)^74^ allow service users to nominate symptoms/aspects of functioning that are concerning them most and to subjectively assess the changes in these symptoms over time following a therapeutic intervention. The potential for the development and use of alternative research measures and designs, such as MYMOP, has not yet been fully embraced by practitioners and policy makers in this field and would make an important move towards providing an evidence-base for miscarriage peer support.

### Randomised controlled trials and their role in evaluation of peer support

The use of RCTs as a methodology to understand the effectiveness of peer support has been heavily critiqued for its reductionist approach.^75 76^ Peer support, by nature, is based on principles of mutual aid, including self-determination and agency, arising spontaneously from community participation, which conflicts with the execution of research designs such as clinical trials, using clinical outcome measures.^77^ Peer support for people who have experienced miscarriage is, itself, a complex system operating within complex contexts of healthcare systems and broader societal structures. Members of such nuanced support groups bring with them their various beliefs, judgments, experiences, personalities, social relationships, intersectional identities and health statuses, making it a replica of the broader complexity.^76^ In the context of peer support after miscarriage, consideration should be afforded to meeting individual peer support needs while considering the wider social determinants of miscarriage. For example, black mothers are disproportionately affected by miscarriage, whereas those with lower socio-economic status are also at higher risk because of factors such as high stress levels resulting from low income or poor working conditions or risky health behaviours.^1^ Further, the risk of major depression in black women who experience early pregnancy loss is twice as high compared with non-black women, whereas women and birthing people with higher socio-economic deprivation have also been reported to be disproportionately vulnerable to mental health problems after miscarriage.^78 79^ As such, it is important to make nuanced considerations of these wider determinants in the development and evaluation of peer support services in the specific context of miscarriage, and utilising measures such as goals-based outcomes could support this.

Given the importance of social context in individual’s experiences of grief and recovery following miscarriage, it may appear inappropriate to consider a research design whereby social context is ignored.^80^ As such, it may be useful to consider peer support as a complex intervention.^81^ In accord with work by Bonell,^82^ who also critiques the use of RCTs to evaluate (complex) public health interventions, we suggest future research designs that account for how intervention components interact and the circumstances that assist interventions to work. This includes considering examining the context of ‘what works, for whom and when’, rather than simply determining that something does work.^83^ In the context of peer support after miscarriage, it is important to consider the implications of social context such as ethnicity, SES, cultural beliefs, social network support and perceived societal stigma on people’s grief and associated recovery outcomes.

High-quality trials with comparable co-designed interventions combined with clear descriptions of mechanisms of change are needed to further investigate peer support efficacy.^84^ Research designs such as realist evaluation which aim to identify underlying generative causal mechanisms that explain how outcomes were caused and how context influence these, situated alongside RCTs, may help to better understand the benefits of peer support for this population.^83^ RCTs may include non-symptom primary outcomes reflective of the values of peer support, in addition to broader process outcomes. A clear definition of peer support and the theoretical underpinning of the intervention, in addition to clearly defined intervention parameters such as adherence (eg, delivery), dose (eg, frequency, duration and intensity) and quality (eg, training) are important in designing a high-quality RCT. Mixed-methods approaches utilising a range of outcome and process evaluation methods are optimal for building a complete picture of the benefits or drawbacks of an intervention.^75 76^

### Cost-effectiveness of peer support

This review found no evidence of the cost-effectiveness of peer support interventions for those who have experienced miscarriage. This is reflected in the wider research context, where there remains a paucity of evidence assessing the economic value of peer support as an intervention.^85^ It is thought that peer support may be less costly to provide, given that the pay scales for peer support workers are considerably lower than those of clinical providers with advanced skills and training.^85^ In the UK context, in an analysis of a mental health service complemented by a peer support service, Simpson *et al*. found increased costs for the intervention group compared with the control group.^86^ While there is argument that peer support has the potential to decrease costly societal outcomes such as utilisation of emergency and inpatient services, there is currently limited and inconclusive evidence to support this.^85 87^ Future research into peer support interventions for those who experience miscarriage should look to include economic evaluations as primary outcomes to determine whether such interventions can help healthcare systems save valuable resources.

### Strengths and limitations

It is commonly perceived that empty reviews, that is, systematic reviews that find no studies eligible for inclusion, do not provide additional information that can be used by clinicians and other decision-makers.^88^ However, we argue that this review is important in that it might help encourage the development and implementation of well-designed interventions and concurrent evaluative studies using alternative approaches and measurement tools as suggested for assessing peer support interventions for parents who have experienced miscarriage. This should look to include research on culturally safe, context-sensitive peer support models that seek to address the highlighted disparities in miscarriage experiences. The review has several strengths including an exhaustive search strategy. This review employed broad inclusion criteria with respect to study design, setting and outcome measures; however, the studies identified were relatively focused in terms of population and peer-support intervention type, reflecting gaps and clustering within the existing evidence base.

The findings should be read with the following limitations in mind. It is possible that unpublished studies or interventions exist such as those carried out in organisations that do not readily publish work. Additionally, the scope of this review was limited to studies published in English language. Studies conducted in other languages might have met the inclusion criteria.

## Conclusions

The loss of a pregnancy through miscarriage can have enduring effects, and the psychological well-being of both women and partners can often be affected. Despite this, there is a paucity of evidence to support peer support interventions for parents after miscarriage. There are wide gaps in knowledge, and current practice recommendations are based on limited data. Given the drive for the inclusion of peer support in new maternal mental health services providing care following loss and the specific mental health support needs of those who have experienced miscarriage, recommendations from this review advocate strongly for targeted intervention research to evaluate peer support interventions. The proposed suggestion to include service user-oriented measures in broader effectiveness evaluations seek to encourage clinicians and decision-makers to bring outcome measures in line with the values of peer support to better demonstrate effectiveness.

## Supplementary material

10.1136/bmjopen-2025-109556 online supplemental file 1

## Data Availability

No data are available.
