# Supplementary material for: Effectiveness of peer support interventions to improve mental health outcomes after miscarriage: a systematic review and call for high-quality evidence
Source: BMJ Open. 2026 Jun 24;16(6):e109556. doi: 10.1136/bmjopen-2025-109556 (PMC13295866; doi:10.1136/bmjopen-2025-109556)
Supplement: online supplemental file 1 [file bmjopen-16-6-s001.docx]

**Supplementary File 1**

**Appendix 1. Search Strategies**

Ovid MEDLINE(R) ALL <1946 to June 5, 2025>

1 peer*.ti,ab,kw.

2 ((lay or layperson* or laypeople or "lay people") adj3 (support* or led or lead* or deliver* or run* or held or direct* or online or "on line" or forum*)).ti,ab,kw.

3 (Parent to parent* adj3 (support* or led or lead* or deliver* or run* or held or direct* or online or "on line" or forum*)).ti,ab,kw.

4 ((Friend* or befriend) adj3 (support* or led or lead* or deliver* or run* or held or direct* or online or "on line" or forum*)).ti,ab,kw.

5 (mutual* adj2 (aid* or support* or help*)).ti,ab,kw.

6 (online adj2 (aid* or support* or help*)).ti,ab,kw.

7 peer group/ or peer influence/

8 Self Help Groups/

9 Social networking/

10 Social support/

11 or/1-10

12 (miscarriage* or miscarry*).ti,ab,kw.

13 (pregnan* adj3 loss*).ti,ab,kw.

14 (fetal loss*).ti,ab,kw.

15 exp Abortion, Spontaneous/

16 exp Perinatal Death/

17 "spontaneous abortion".ti,ab,kw.

18 "perinatal death".ti,ab,kw.

19 or/12-18

20 11 and 19

21 limit 20 to english language

22 limit 21 to yr="1990 -Current"

**CINAHL**

S1 TI peer* OR AB peer*

S2 TI ( ((lay or layperson* or laypeople or "lay people") N3 (support* or led or lead* or deliver* or run* or held or direct* or online or "on line" or forum*)) ) OR AB ( ((lay or layperson* or laypeople or "lay people") N3 (support* or led or lead* or deliver* or run* or held or direct* or online or "on line" or forum*)) )

S3 TI ( (Parent to parent* N3 (support* or led or lead* or deliver* or run* or held or direct* or online or "on line" or forum*)) ) OR AB ( (Parent to parent* N3 (support* or led or lead* or deliver* or run* or held or direct* or online or "on line" or forum*)) )

S4 TI ( ((Friend* or befriend) N3 (support* or led or lead* or deliver* or run* or held or direct* or online or "on line" or forum*)) ) OR AB ( ((Friend* or befriend) N3 (support* or led or lead* or deliver* or run* or held or direct* or online or "on line" or forum*)) )

S5 TI ( (mutual* N2 (aid* or support* or help*)) ) OR AB ( (mutual* N2 (aid* or support* or help*)) )

S6 TI ( (online N2 (aid* or support* or help*)) ) OR AB ( (online N2 (aid* or support* or help*)) )

S7 (MH "Peer Group") OR (MH "Peer Counseling")

S8 (MH "Support Groups")

S9 (MH "Social Networks")

S10 (MH "Support, Social+")

S11 S1 OR S2 OR S3 OR S4 OR S5 OR S6 OR S7 OR S8 OR S9 OR S10

S12 TI ( (miscarriage* or miscarry*) ) OR AB ( (miscarriage* or miscarry*) )

S13 TI ((pregnan* N3 loss*) OR ("fetal loss") OR ("spontaneous abortion") OR ("perinatal death")) OR AB ((pregnan* N3 loss*) OR ("fetal loss") OR ("spontaneous abortion") OR ("perinatal death"))

S14 (MH "Abortion, Spontaneous+")

S15 (MH "Perinatal Death")

S16 S12 OR S13 OR S14 OR S15

S17 S11 AND S16

S18 S11 AND S16

S19 S11 AND S16

Narrow by Language: - english

Limiters - Publication Year: 1990-2024

**APA PsycINFO**

S1 TI peer* OR AB peer*

S2 TI ( ((lay or layperson* or laypeople or "lay people") N3 (support* or led or lead* or deliver* or run* or held or direct* or online or "on line" or forum*)) ) OR AB ( ((lay or layperson* or laypeople or "lay people") N3 (support* or led or lead* or deliver* or run* or held or direct* or online or "on line" or forum*)) )

S3 TI ( (Parent to parent* N3 (support* or led or lead* or deliver* or run* or held or direct* or online or "on line" or forum*)) ) OR AB ( (Parent to parent* N3 (support* or led or lead* or deliver* or run* or held or direct* or online or "on line" or forum*)) )

S4 TI ( ((Friend* or befriend) N3 (support* or led or lead* or deliver* or run* or held or direct* or online or "on line" or forum*)) ) OR AB ( ((Friend* or befriend) N3 (support* or led or lead* or deliver* or run* or held or direct* or online or "on line" or forum*)) )

S5 TI ( (mutual* N2 (aid* or support* or help*)) ) OR AB ( (mutual* N2 (aid* or support* or help*)) )

S6 TI ( (online N2 (aid* or support* or help*)) ) OR AB ( (online N2 (aid* or support* or help*)) )

S7 (DE "Peer Support") OR (DE "Peer Counseling")

S8 DE "Support Groups" OR DE "Online Support Groups"

S9 DE "Social Networks" OR DE "Online Social Networks"

S10 DE "Social Support"

S11 S1 OR S2 OR S3 OR S4 OR S5 OR S6 OR S7 OR S8 OR S9 OR S10

S12 TI ( (miscarriage* or miscarry*) ) OR AB ( (miscarriage* or miscarry*) )

S13 TI ((pregnan* N3 loss*) OR ("fetal loss") OR ("spontaneous abortion") OR ("perinatal death")) OR AB ((pregnan* N3 loss*) OR ("fetal loss") OR ("spontaneous abortion") OR ("perinatal death"))

S14 DE "Spontaneous Abortion"

S15 S12 OR S13 OR S14

S16 S11 AND S15

S17 S11 AND S15

S18 S11 AND S15

Limiters - Published: 19900101-20241231

Narrow by Language: - english

**Embase**

Embase <1974 to 2025 June 5>

1 peer*.ti,ab,kw. 178776

2 ((lay or layperson* or laypeople or "lay people") adj3 (support* or led or lead* or deliver* or run* or held or direct* or online or "on line" or forum*)).ti,ab,kw. 1638

3 (Parent to parent* adj3 (support* or led or lead* or deliver* or run* or held or direct* or online or "on line" or forum*)).ti,ab,kw. 117

4 (Parent to parent* adj3 (support* or led or lead* or deliver* or run* or held or direct* or online or "on line" or forum*)).ti,ab,kw. 117

5 ((Friend* or befriend) adj3 (support* or led or lead* or deliver* or run* or held or direct* or online or "on line" or forum*)).ti,ab,kw. 6629

6 (mutual* adj2 (aid* or support* or help*)).ti,ab,kw. 3292

7 (online adj2 (aid* or support* or help*)).ti,ab,kw. 4524

8 peer group/ 32247

9 self help/ 15136

10 social network/ or social support/ 144348

11 1 or 2 or 3 or 4 or 5 or 6 or 7 or 8 or 9 or 10 343156

12 (miscarriage* or miscarry*).ti,ab,kw. 33446

13 (pregnan* adj3 loss*).ti,ab,kw. 17801

14 fetal loss*.ti,ab,kw. 6523

15 "spontaneous abortion".ti,ab,kw. 12155

16 "perinatal death".ti,ab,kw. 4865

17 spontaneous abortion/ 53930

18 perinatal death/ 5341

19 12 or 13 or 14 or 15 or 16 or 17 or 18 86903

20 11 and 19 895

21 limit 20 to (english language and yr="1990 -Current") 848

**Cochrane CENTRAL**

ID Search Hits

#1 (peer*):ti,ab,kw

#2 (((lay or layperson* or laypeople or "lay people") near/3 (support* or led or lead* or deliver* or run* or held or direct* or online or "on line" or forum*))):ti,ab,kw

#3 ((Parent to parent* near/3 (support* or led or lead* or deliver* or run* or held or direct* or online or "on line" or forum*))):ti,ab,kw

#4 ((Friend* or befriend) near/3 (support* or led or lead* or deliver* or run* or held or direct* or online or "on line" or forum*))

#5 ((mutual* near/2 (aid* or support* or help*))):ti,ab,kw

#6 ((online near/2 (aid* or support* or help*))):ti,ab,kw

#7 MeSH descriptor: [Peer Group] this term only

#8 MeSH descriptor: [Peer Influence] this term only

#9 MeSH descriptor: [Self-Help Groups] this term only

#10 MeSH descriptor: [Social Networking] this term only

#11 MeSH descriptor: [Social Support] this term only

#12 (48-#11)

#13 ((miscarriage* or miscarry*)):ti,ab,kw

#14 ((pregnan* near/3 loss*)):ti,ab,kw

#15 (fetal NEXT loss*):ti,ab,kw

#16 MeSH descriptor: [Abortion, Spontaneous] this term only

#17 MeSH descriptor: [Perinatal Death] this term only

#18 ("spontaneous abortion"):ti,ab,kw

#19 ("perinatal death"):ti,ab,kw

#20 (11-#19)

#21 #12 AND #20

**Web of Science**

Peer* OR ((lay or layperson* or laypeople or "lay people") NEAR/3 (support* or led or lead* or deliver* or run* or held or direct* or online or "on line" or forum*)) OR (Parent to parent* NEAR/3 (support* or led or lead* or deliver* or run* or held or direct* or online or "on line" or forum*)) OR ((Friend* or befriend) NEAR/3 (support* or led or lead* or deliver* or run* or held or direct* or online or "on line" or forum*)) OR (mutual* NEAR/2 (aid* or support* or help*)) OR (online NEAR/2 (aid* or support* or help*)) OR "self-help group*" OR "social networking" OR "social support"

miscarriage* OR miscarry* OR pregnan* NEAR/3 loss* OR " fetal loss*" OR "spontaneous abortion" OR "perinatal death"

Limit to 1990, English language

**LENS.org**

( Title: ( miscarriage OR ( "pregnancy loss" OR ( "fetal loss" OR ( "spontaneous abortion" OR "perinatal death" ) ) ) ) OR ( Abstract: ( miscarriage OR ( "pregnancy loss" OR ( "fetal loss" OR ( "spontaneous abortion" OR "perinatal death" ) ) ) ) OR ( Keyword: ( miscarriage OR ( "pregnancy loss" OR ( "fetal loss" OR ( "spontaneous abortion" OR "perinatal death" ) ) ) ) OR Field of Study: ( miscarriage OR ( "pregnancy loss" OR ( "fetal loss" OR ( "spontaneous abortion" OR "perinatal death" ) ) ) ) ) ) ) AND ( Title: ( "peer support" OR ( "social support" OR ( "social networking" OR ( "social networks" OR "support groups" ) ) ) ) OR ( Abstract: ( "peer support" OR ( "social support" OR ( "social networking" OR ( "social networks" OR "support groups" ) ) ) ) OR Keyword: ( "peer support" OR ( "social support" OR ( "social networking" OR ( "social networks" OR "support groups" ) ) ) ) ) )

Limit to 1990-, journal article , preprint , dissertation

**British Nursing Index**

1. tiab(peer*) OR tiab(((lay OR layperson* OR laypeople OR "lay people") PRE/3 (support* OR led OR lead* OR deliver* OR run* OR held OR direct* OR online OR "on line" OR forum*))) OR tiab((Parent to parent* P3 (support* OR led OR lead* OR deliver* OR run* OR held OR direct* OR online OR "on line" OR forum*))) OR tiab(((Friend* OR befriend) P3 (support* OR led OR lead* OR deliver* OR run* OR held OR direct* OR online OR "on line" OR forum*))) OR tiab((mutual* P2 (aid* OR support* OR help*))) OR MAINSUBJECT.EXACT("Social support") OR MAINSUBJECT.EXACT("Social networks") OR MAINSUBJECT.EXACT("Support groups")
2. tiab(miscarriage* OR miscarry*) OR tiab(pregnan* P3 loss*) OR tiab("fetal loss*") OR tiab("spontaneous abortion") OR tiab("perinatal death") OR MAINSUBJECT.EXACT("Miscarriage")
3. Limit to English, 1990-

**HMIC Health Management Information Consortium <1979 to May 2024>**

1 peer*.ti,ab.

2 ((lay or layperson* or laypeople or "lay people") adj3 (support* or led or lead* or deliver* or run* or held or direct* or online or "on line" or forum*)).ti,ab.

3 (Parent to parent* adj3 (support* or led or lead* or deliver* or run* or held or direct* or online or "on line" or forum*)).ti,ab.

4 ((Friend* or befriend) adj3 (support* or led or lead* or deliver* or run* or held or direct* or online or "on line" or forum*)).ti,ab.

5 (mutual* adj2 (aid* or support* or help*)).ti,ab.

6 (online adj2 (aid* or support* or help*)).ti,ab.

7 exp Support groups/ or exp Peer groups/

8 exp Self help groups/

9 exp Social networking/

10 exp Social support/

11 or/1-10

12 (miscarriage* or miscarry*).ti,ab.

13 (pregnan* adj3 loss*).ti,ab.

14 fetal loss*.ti,ab.

15 "spontaneous abortion".ti,ab.

16 "perinatal death".ti,ab.

17 exp Miscarriages/

18 exp Perinatal mortality/

19 or/12-18

20 11 and 19

**ProQuest Dissertations and Theses**

1. tiab(peer*) OR tiab(((lay OR layperson* OR laypeople OR "lay people") PRE/3 (support* OR led OR lead* OR deliver* OR run* OR held OR direct* OR online OR "on line" OR forum*))) OR tiab((Parent to parent* P3 (support* OR led OR lead* OR deliver* OR run* OR held OR direct* OR online OR "on line" OR forum*))) OR tiab(((Friend* OR befriend) P3 (support* OR led OR lead* OR deliver* OR run* OR held OR direct* OR online OR "on line" OR forum*))) OR tiab((mutual* P2 (aid* OR support* OR help*)))
2. tiab(miscarriage* OR miscarry*) OR tiab(pregnan* P3 loss*) OR tiab("fetal loss*") OR tiab("spontaneous abortion") OR tiab("perinatal death")
3. Limit to English, 1990-

**Google Scholar targeted search**

allintitle: miscarriage peer OR "social support" OR "social networks" OR "social networking"

allintitle: miscarry peer OR "social support" OR "social networks" OR "social networking"

allintitle: "pregnancy loss" peer OR "social support" OR "social networks" OR "social networking"

allintitle: "fetal loss" peer OR "social support" OR "social networks" OR "social networking"

allintitle: "spontaneous abortion" peer OR "social support" OR "social networks" OR "social networking"

allintitle:"perinatal death" peer OR "social support" OR "social networks" OR "social networking"

Limited to 1990-

**Appendix 2. Details of excluded studies using comparative designs**

| **Author** | **Country** | **Study Design** | **Population** | **Intervention** | **Comparator** | **Outcomes**  **(Measures used)** | **Reason for Exclusion** |
| --- | --- | --- | --- | --- | --- | --- | --- |
| Hung et al. 2023(42) | Taiwan | RCT* with a prospective pre-post test experimental design. | Recurrent miscarriage | Website -  12-week intervention with access to online forum | Control group – routine care | Perceived stress, depression, sleep quality and social support | Wrong intervention – not peer support (website – does include an online forum but as adjunct to wider intervention) |
| Sun et al. 2018(43) | China | RCT* | Pregnant women with foetal abnormalities requiring pregnancy termination (termination for medical reasons; TFMR) | Family-support programme  6-week intervention | Control group – routine care | Family support, depression, post-traumatic stress symptoms | Wrong intervention – not peer support (healthcare professional-led (nurse) educational support) |
| Bailey et al. 2015(44) | UK | Feasibility RCT* | Recurrent miscarriage | Positive Reappraisal Coping Intervention  (self-management psychoeducation) | Control group – routine care | Quality of life – depression (Hospital Anxiety and Depression Scale) | Wrong intervention – not peer support (self-management) |
| Gold et al. 2021(45) | USA | Feasibility RCT* | Bereaved mothers - given birth to a stillborn baby or had an infant death in the first month of life | Online support  6-week intervention | Participants randomised into;  1)online support via anonymous BabyCenter.com loss support group  2)closed Facebook loss group  3)control group | E-mail survey, PHQ-9 (depression), online satisfaction  Michigan Mothers Survey (additional demographics at time of loss) | Wrong population – given birth to a stillborn baby or had an infant death in the first month of life |
| Rajan & Oakley 1993(46) | UK | RCT* | Pregnancy after loss | Social support intervention – minimum 3 home visits and 2 telephone calls between visits by research midwife |  | Emotional wellbeing.  Social Support and Pregnancy Outcome (SSPO) Study | Wrong population – pregnancy after loss  Wrong intervention – not peer support (healthcare professional-led) |
| Johnson & Langford 2015(47) | USA | RCT* | Women experiencing pre-20-week loss and receiving care in Emergency Department | Healthcare professional delivered intervention in hospital-setting | Control group – routine care | Grief, coping and despair measured 2 weeks after miscarriage | Wrong intervention – not peer support (healthcare professional-led) |
| Neal 2023(49) | USA | Longitudinal cohort study (retrospective review of medical records) | Pregnancy loss at any gestation | Perinatal bereavement treatment programme (Butterfly’s Embrace Family Support Programme (BEFSP) delivered by healthcare professionals. | No comparator – retrospective longitudinal data collected from individuals who accessed the service | Edinburgh Postnatal Depression Scale | Wrong intervention – not peer support (healthcare professional-led) |
| DiMarco et al. 2001(50) | USA | A cross-sectional, retrospective, two-group research design was used to determine if there were differences in grief reactions between parents who attended a support group versus parents who had not. | Pregnancy loss at any gestation | Support group (peers) – minimum attendance once.  Co-facilitated by primary author or nurse/patient and chaplain.  Met once per month for 2 hours and attended by 8-12 people. | Those who had attended a support group v. those who had not. | Hogan Grief Reaction Checklist Grief Scale | Wrong population – mixed sample and unable to separate out miscarriage-specific data |
| Loughnan et al. 2024(48) | Australia | RCT* | Stillbirth (defined as loss <20 weeks gestation or neonatal death | 8-week modular intervention (online) including lived experience | Control group – routine care | Psychological distress – Kessler Psychological Distress Scale  Grief, anxiety, depression, decisional regret about healthcare decisions, prolonged grief, resilience | Wrong population – mixed sample and unable to separate out miscarriage-specific data    Wrong intervention – not peer support |

Abbreviations: RCT* Randomised Controlled Trial
