# Supplementary material for: Effectiveness of peer support interventions to improve mental health outcomes after miscarriage: a systematic review and call for high-quality evidence
Source: BMJ Open. 2026 Jun 24;16(6):e109556. doi: 10.1136/bmjopen-2025-109556 (PMC13295866; doi:10.1136/bmjopen-2025-109556)
Supplement: Supplementary file 2 [file bmjopen-2025-109556.reviewer_comments.pdf]

PEER REVIEW HISTORY

BMJ Open publishes all reviews undertaken for accepted manuscripts. Reviewers are asked to complete a checklist review form and are provided with free text boxes to elaborate on their assessment. These free text comments are reproduced below.

ARTICLE DETAILS

Title (Provisional)

The effectiveness of peer support interventions to improve mental health outcomes after miscarriage: a systematic review and call for high quality evidence

Authors

Burton, Leanne; Charles, Juanita; Cherry, M. Gemma; Corcoran, Rhiannon; Hassan, Shaima; Hill, Ruaraidh; Little, Sarah; Maden, Michelle; Mulholland, Helen; Perkins, Elizabeth; Slade, Pauline; Wallis, Selina; Marshall, Paul

---

VERSION 1 - REVIEW

**Reviewer**	**1**
**Name**	**Sharip, Shalisah**
**Affiliation**	**Universiti Kebangsaan Malaysia Medical Centre**
**Date**	**23-Sep-2025**
**COI**	**None**

Dear Authors,

Below are comments on your manuscript

1. State the gap of the study clearly

2. Brief add information on peer support intervention.how this intervention applicable to support grief mum .

3. I am wondering about exclusion criteria and besides the two author extract and review the articles..do they seek third reviewer to verify decision on exclude the paper

4. Since this review provide negative finding it is good to add some narrative explanation on selective 7 randomised control group article

---

**Reviewer**	**2**
**Name**	**Yasmin, Haleema**
**Affiliation**	**Jinnah Post Granduate Medical Center, Obstetrics & Gynaecology**

**Date**	**01-Jan-2026**
**COI**	**None**

well written systematic review, highlighting the gaps present in interventions for peer-support after miscarriage.

**Reviewer**	**3**
**Name**	**Singh, Megha**
**Affiliation**	**University of Birmingham, Applied health research**
**Date**	**21-Apr-2026**
**COI**	**None**

No furtehr comments,happy with revisions made

VERSION 1 - AUTHOR RESPONSE

**Reviewer 1 Comments**	
1. State the gap of the study clearly	Thank you for this comment. Along with the clarification of the importance of differentiating the support needs of pregnancy loss at later gestations from those experienced after early pregnancy loss, as requested by the Associate Editor, we have now also stated clearly the gap that the study seeks to address (page 8, line 194-197).
2. Brief add information on peer support intervention.how this intervention applicable to support grief mum.	Thank you for this suggestion. We agree that clarifying the nature and relevance of peer support is important. This information is already outlined in the Introduction.<br>To improve clarity, we have also slightly revised this section to make the applicability to grief more explicit (page 7, line 178-181).
3. I am wondering about exclusion criteria and besides the two author extract and review the articles..do they seek third reviewer to verify decision on exclude the paper	Thank you for requesting additional clarity on this. We had added in that any disagreements were discussed with a third reviewer (page 10, line 246-247).
4. Since this review provide negative finding it is good to add some narrative explanation on selective 7 randomised control group article	Thank you for this constructive suggestion. We agree that providing additional context on studies identified but not fully meeting the inclusion criteria strengthens the interpretation of our findings. We had

	already included a brief description of these trials; however, in response to this comment, we have further expanded this section to provide a clearer context of the current evidence base and further highlight the gap in rigorously evaluated peer support interventions (page 12, line 285-289).
**Reviewer 2 Comments**	
well written systematic review, highlighting the gaps present in interventions for peer-support after miscarriage.	We are grateful to the reviewer for their positive feedback and for recognising the contribution of this review in identifying gaps in peer-support after miscarriage.
**Reviewer 3 Comments**	
No further comments, happy with revisions made	We thank the reviewer for their positive assessment of the manuscript.
